# Transdiagnostic symptom networks in adolescent psychopathology: A longitudinal panel network analysis

**DOI:** 10.1002/jcv2.70118

**Published:** 2026-04-06

**Authors:** Xenia A. Häfeli, Larisa Morosan, Anja Hirsig, Stefanie J. Schmidt

**Affiliations:** ^1^ Division of Clinical Child and Adolescent Psychology University of Bern Bern Switzerland

**Keywords:** adolescence, early detection, mental health, network, prevention, subclinical, transdiagnostic

## Abstract

**Background:**

Understanding symptom patterns of emerging psychopathology is essential for early detection and intervention. Network analysis offers a promising approach by conceptualizing emerging psychopathology as dynamic interactions between symptoms over time. This study aims to explore the structure of a transdiagnostic symptom network in adolescents.

**Methods:**

A one‐year prospective study was conducted with a general population sample of 1186 Swiss adolescents with no current or past mental disorders (data collection: February 2021‐March 2024; age 11–17 years (*M* = 14.19, SD = 1.90); 56.5% female). Participants completed online self‐report questionnaires on 29 symptoms across seven mental disorders at five time points. Panel Graphical Vector Autoregression (Panel GVAR) models were used to estimate contemporaneous and temporal symptom networks. Centrality measures, including node expected influence (EI), bridge expected influence (BEI), bridge betweenness (BB), in‐strength, and out‐strength, were computed to characterize the network.

**Results:**

In the contemporaneous network, anxiety, depressive, and eating disorder symptoms, particularly excessive worry (EI = 1.08), body dissatisfaction (EI = 1.03), and low self‐esteem (EI = 1.01), exhibited the highest EI. Irritability (BEI = 0.80/BB = 0.58) and low self‐esteem (BEI = 0.57/BB = 0.50) acted as important bridge symptoms within the network. In the temporal network, uncontrollable worry (*β* = 0.71) predicted anxiety and depressive symptoms. Body dissatisfaction (*β* = 1.09) and weight concerns (*β* = 1.04) predicted symptoms across various disorder domains, including anxiety, depressive, psychotic, bipolar, and obsessive‐compulsive symptoms. Depressive symptoms were primarily influenced by other symptoms.

**Conclusion:**

Worry, body dissatisfaction and weight concerns play a pivotal role in emerging psychopathology by linking multiple disorder domains and shaping symptom dynamics over time. These findings may enhance early detection by identifying high‐risk adolescents and may inform prevention strategies, such as interventions targeting excessive worry and negative body image, to reduce symptom escalation and improve long‐term mental health outcomes.

## Key Points

**What's known?**

- Network modeling offers significant benefits in understanding and addressing emerging psychopathology in adolescence. Psychopathological networks have been used to model symptom associations in single or few mental disorders.

**What's new?**

- A network of symptoms from various mental disorders commonly emerging in adolescence revealed worry, body dissatisfaction, and weight concerns as central symptoms, with high influence on symptoms both within and across time. Irritability and low self‐esteem were important bridge symptoms, connecting multiple disorder domains.

**What's relevant?**

- These findings emphasize the need for early interventions targeting central symptoms as they may drive symptom progression. Bridge symptoms may be important transdiagnostic targets as they may reduce the progression of symptoms across mental disorder domains and may thereby prevent the development of comorbidity.

## INTRODUCTION

Adolescence is a critical developmental period marked by significant cognitive, emotional, and social changes, with the overall peak age of onset around 14–15 years (McGrath et al., 2023; Solmi et al., 2022). Notably, several mental disorders associated with social‐emotional and self‐regulatory processes show age‐specific increases in incidence rates (Rapee et al., 2019): social anxiety and conduct disorder commonly rise in early adolescence, while others increase more prominently in mid‐to‐late adolescence (e.g., eating and depressive disorders, obsessive‐compulsive disorder (OCD), substance use disorder, bipolar disorder, psychotic disorders) (Kessler et al., 2005; McGrath et al., 2023). Subclinical symptoms are early signs of mental health difficulties (e.g., low or irritable mood, fearfulness) that fall short of diagnostic thresholds, as they do not meet the required number, frequency, or intensity of symptoms (Lee et al., 2019; Rodríguez et al., 2012). While most of these symptoms remain subclinical over time or remit (Schreuder et al., 2021), they may nevertheless be associated with functional impairment (Lustig et al., 2023), and a subset of them progresses to a full‐threshold mental disorder (Iorfino et al., 2019). Despite this, adolescents have the lowest access to mental health care across the lifespan (McGorry & Mei, 2018), highlighting the need to detect individuals in need of care as early as possible (Asselmann et al., 2018; Solmi et al., 2022). To this aim, a community‐based approach shows promise to detect individuals with early signs of emerging psychopathology who may not be actively seeking help and might otherwise go unnoticed (Fusar‐Poli et al., 2021; Murray et al., 2021).

In emerging psychopathology, subclinical symptoms from different diagnostic entities often co‐occur and interact with each other over time (Wigman et al., 2013). The network approach seems especially appealing to study emerging psychopathology as it assumes that psychopathology results from self‐reinforcing symptom interactions rather than a single underlying cause and is best understood as a dynamic system of interconnected symptoms spanning and linking multiple mental disorder domains (Borsboom, 2017). Therefore, it aligns well with the transdiagnostic approach to mental health, which also emphasizes the role of symptom interactions and shared mechanisms underlying several mental disorders (Dalgleish et al., 2020).

In psychopathological networks, symptoms are represented as nodes, and the associations between them as edges (Borsboom & Cramer, 2013; Jordan et al., 2020). Centrality metrics reveal the role and influence of specific nodes within the network structure. Nodes with high centrality exhibit numerous or strong associations to other nodes and potentially perpetuate mental health problems by facilitating the spread of activation between connected nodes (Blanken et al., 2018). Nodes with high bridge centrality link multiple disorder domains (i.e., diagnostic categories), potentially serving as pathways through which activation from one domain can spread to another (Jones et al., 2021). Identifying central and bridge symptoms can enhance early detection efforts, as central symptoms may act as early indicators of emerging psychopathology, while bridge symptoms may explain the co‐occurrence of different mental disorder domains (Borsboom & Cramer, 2013; Jones et al., 2021).

Several studies have used network analysis to examine the structure of subclinical symptoms in adolescent psychopathology using cross‐sectional data (Boschloo et al., 2016; Groen et al., 2019; Imperiale et al., 2023; Ródenas‐Perea et al., 2025; Wang et al., 2021). They have consistently shown that symptoms within the same mental disorder domain exhibit stronger associations than those across different domains. Additionally, depressive symptoms have consistently been identified as being central (Boschloo et al., 2016; Wang et al., 2021). However, cross‐sectional studies cannot capture the predictive relationships between symptoms. Therefore, temporal networks are needed to model symptom interactions across multiple time points (Epskamp, 2020). Previous studies in adolescent general population samples have used temporal networks to examine the predictive relationships of symptoms within single disorders, such as depression (Hoffart et al., 2023), substance abuse (Freichel, Pfirrmann, et al., 2023a), eating disorders (Zhang et al., 2024), and anxiety (Abend et al., 2021). Studies including multiple disorder domains in adolescent samples have primarily focused on temporal networks on disorder‐level. Therein, each node represents a diagnostic entity rather than an individual symptom. For example, “depressive problems” have been identified as a central bridge node predicting other internalizing symptoms (Freichel et al., 2024; Liu et al., 2023), while “aggressive behaviors” served as central bridge node between internalizing and externalizing problems (Liu et al., 2023). Similarly, “hyperactivity/inattention” has been found to link “emotional problems”, “peer problems,” “conduct problems”, and “prosociality” (Speyer et al., 2022). While disorder‐level networks reveal broad patterns, such as depression predicting anxiety (Freichel et al., 2024), temporal symptom‐level networks are crucial for identifying the specific symptoms involved (Jordan et al., 2020).

Previous temporal network studies have examined emerging psychopathology in adult general population and clinical samples (e.g., Arribas et al., 2025; Odenthal et al., 2023; Peng et al., 2024). However, to our knowledge, only two studies have investigated transdiagnostic temporal symptom‐level associations in adolescent general population samples (Black et al., 2022; Funkhouser et al., 2021). The first study examined temporal associations between symptoms from internalizing, externalizing, and attention domains in adolescents aged 9–12 years (Funkhouser et al., 2021). Depressed mood, inattention, and worry were the most predictive symptoms, while threats of violence and destructiveness were the symptoms most strongly influenced by others. The relationship between depressed mood and worthlessness was one of the strongest bidirectional associations in the network. The second study analyzed temporal relationships between internalizing symptoms, well‐being, and inter‐/intrapersonal aspects in adolescents aged 11–15 years (Black et al., 2022). Worry was again identified as the most central symptom. However, while both studies recruited general population samples, they did not explicitly exclude individuals with a current or past mental disorder. Therefore, it remains unclear whether they examined subclinical symptoms of emerging psychopathology or subclinical symptoms within manifest mental disorders. Thus, in line with an indicated prevention framework (Fusar‐Poli et al., 2021), our study excluded participants with neurodevelopmental and other previous mental disorders to specifically investigate mechanisms underlying newly emerging psychopathology in adolescence, rather than persistent developmental conditions. Moreover, while both recent studies focused on internalizing and externalizing symptoms, they did not assess symptoms linked to eating disorders, bipolar disorder, psychotic disorders, or OCD, which typically begin in adolescence (Solmi et al., 2022). To fully capture the complexity and heterogeneity of psychopathology, it is essential to include a broader range of symptoms across various mental disorders. Further, both studies relied on only three assessment points over 1 year (Funkhouser et al., 2021) and 2 years (Black et al., 2022). Shorter time lags might offer a more precise understanding of how symptoms emerge and reinforce each other (Bringmann et al., 2023; Helmich et al., 2021).

To extend previous findings, this study applied novel network modeling techniques to identify central and bridge symptoms across multiple mental disorder domains in a general population sample of adolescence without any past or current mental disorder. Based on previous studies (Black et al., 2022; Funkhouser et al., 2021) and staging models of emerging psychopathology (Hickie et al., 2013) it was assumed that in particular anxiety and depressive symptoms may be relevant core and bridge symptoms in a transdiagnostic network.

## METHODS

### Sample

Participants were recruited as part of a 1‐year prospective, naturalistic epidemiological study investigating trajectories, patterns, and early warning signs of subclinical symptoms and transdiagnostic mechanisms in emerging psychopathology (EMERGE‐study; Häfeli et al., 2024; SNSF: PCEGP1‐186913). The EMERGE‐study included a general population sample of Swiss adolescents meeting the following criteria: (1) aged 11–17 years; (2) fluent in German; (3) residing in a German‐speaking canton of Switzerland; and (4) having access to the Internet. The age range of 11–17 years was chosen to cover the developmental period during which the onset of most adolescent mental disorders occurs (McGrath et al., 2023) ensuring that the study captures the initial appearance, co‐occurrence, and interaction of subclinical symptoms across various mental disorders. Due to the focus of the study on emerging psychopathology, participants were excluded if they had a current and/or past diagnosis of a mental disorder according to the “Diagnostic Interview for Mental Disorders for Children and Adolescents” (Kinder‐DIPS; Margraf et al., 2017; Schneider et al., 2017) or a known developmental disorder as defined by the Diagnostic and Statistical Manual of Mental Disorders (DSM‐5; American Psychiatric Association, 2013). Adolescents with a current or past diagnosis of specific phobia were not excluded from the study due to the typically transient nature of specific phobias during adolescence (Benjet et al., 2012; Wardenaar et al., 2017).

### Procedure

The study received ethical approval from the Cantonal Ethics Committee (KEK) Bern (ID 2020‐02108). Data collection took place between February 2021 and March 2024. Contact information about potential participants, including names, addresses, and, if available, phone numbers, was provided by the Federal Statistical Office. The selection process was random but stratified based on age, gender, and regionalization levels (rural, intermediate, and urban areas, as defined by the Federal Statistical Office). Personalized invitation letters were sent to potential participants and their parents, followed by phone calls to provide the study information and to ask for verbal and written informed consent. For adolescents < 14 years, parents or legal guardians provided written electronic consent, while the adolescents gave oral assent. Adolescents ≥ 14 years provided oral and written electronic consent themselves (Häfeli et al., 2024). Once written consent was obtained, diagnostic telephone interviews were carried out to assess if participants fulfilled the diagnostic criteria for a past or current mental disorder. Eligible participants then received a personalized link via email to complete online self‐report questionnaires at five time points: baseline, 3‐month, 6‐month, 9‐month, and 12‐month follow‐up. The 12‐month follow‐up period with 3‐month assessment intervals was chosen based on prior longitudinal research on subclinical symptom trajectories in adolescents (Haller et al., 2014; Lee et al., 2019). Five assessments over 1 year allow the detection of short‐term symptom changes while balancing methodological rigor and study feasibility (Schreuder et al., 2021; Teague et al., 2018). All collected data was securely stored using a web‐based platform (Research Electronic Data Capture, REDCap; Harris et al., 2009, 2019). Participants received vouchers for each completed questionnaire and interview.

### Measures

In this study, 29 subclinical symptoms corresponding to seven mental disorders with a typical onset during adolescence (i.e., anxiety disorders, conduct disorders, depressive disorders, eating disorders, OCD, psychotic disorders, and bipolar disorders (Solmi et al., 2022)) were assessed through online questionnaires. An overview of the instruments is provided in Table 1.

**TABLE 1:** Overview of assessment instruments.

Mental disorder domain	Measures
Anxiety disorders	The “Generalized Anxiety Disorder Screener” (GAD‐7; Löwe et al., 2007; Spitzer et al., 2006), is a 7‐item questionnaire assessing general anxiety symptoms on a 4‐point Likert scale from *Not at all* (0) to *Nearly every day* (3). The instrument has demonstrated good psychometric properties in adolescents (Casares et al., 2024; Tiirikainen et al., 2019).
Bipolar disorder	The “Altman Self‐Rating Mania Scale” (ASRM; Altman et al., 1997) is a 5‐item questionnaire assessing bipolar symptoms on a 5‐point Likert scale from *Not present* (0) to *Present to severe degree* (4). The instrument revealed good psychometric properties in clinical and healthy samples (E. Altman et al., 2001; Meyer et al., 2020).
Conduct disorders	The subscale “conduct problems” (5 items) from the “Strength and Difficulties Questionnaire, Self‐report” (SDQ‐S; Goodman, 2001) was used to assess conduct problems. The items are rated on a 2‐point Likert scale from *not true* (0) to *certainly true* (2). The German version showed moderate to good psychometric properties (Becker et al., 2018; Goodman, 2001).
Depressive disorders	The “Patient Health Questionnaire‐9 for Adolescents” (PHQ‐A; Johnson et al., 2002) is a 9‐item questionnaire assessing the frequency of depressive symptoms on a 4‐point Likert scale from *Not at all* (0) to *Nearly every day* (3). The PHQ‐A revealed good psychometric properties in adolescents (Richardson et al., 2010).
Eating disorders	The “Child Eating Disorder Examination‐Questionnaire” (ChEDE‐Q8; Kliem et al., 2017) is an 8‐item questionnaire assessing eating disorder psychopathology related to anorexia nervosa, bulimia nervosa, and binge‐eating on a 6‐point scale from 0 to 6. The ChEDE‐Q8 showed good psychometric properties and is suitable for non‐clinical as well as clinical populations (Kliem et al., 2017).
Obsessive‐compulsive disorder	The “Short Obsessive‐Compulsive Disorder Screener” (SOCS; Piqueras et al., 2015) is a 6‐item questionnaire assessing obsessive‐compulsive symptoms on a 3‐point Likert scale from *No* (0) to *A lot* (3). It is considered a well‐established instrument to assess obsessive‐compulsive disorder in adolescent clinical samples and revealed moderate to good psychometric properties (Piqueras et al., 2015; Uher et al., 2007).
Psychotic disorders	The “Community Assessment of Psychic Experiences—Positive” (CAPE‐P15; Capra et al., 2017) is a 15‐item questionnaire measuring positive psychotic symptoms on two subscales. The first subscale measures the frequency of symptoms on a 4‐point Likert scale from *Never* (0) to *Very often* (3). The second subscale measures the degree of distress caused by any endorsed experience from *Not distressed (0)* to *Very distressed (3)*. For this study, the frequency and distress subscales were combined by multiplying the frequency and the distress score with each other (Jaya et al., 2021). The CAPE‐P15 showed good psychometric properties in an adolescent general population sample (Núñez et al., 2021).

### Statistical analysis

All analyses were conducted using R statistical software, version 4.4.0 (R Core Team, 2024). To decrease model complexity and to improve interpretability of the results, node selection was carefully guided by both statistical and theoretical considerations. First, we excluded the variables showing low variability, as they may contribute little to the network structure because there is insufficient variation to establish meaningful relationships with other nodes. Including such nodes may even introduce spurious connections or noise (Epskamp et al., 2018; Fried & Cramer, 2017). A scaled variance threshold of 5% was applied as a cutoff, following established procedures in variability analysis (Gelman et al., 2020; Sheskin, 2003). Symptoms below this threshold were excluded unless clear theoretical justification supported their inclusion. Second, based on theoretical considerations, somatic symptoms were excluded from the analysis as the primary aim of the study was to examine the network structure of psychological symptoms. Thus, of the 66 originally assessed symptoms (see Supporting Information S1: Appendix S1, Table S1), 29 symptoms corresponding to seven mental disorders were retained (see Table 2).

**TABLE 2:** Variables and description of each item included in the network (*N* = 1186).

Node	Variable	Domain and item description
Anxiety disorders		
1	GAD 1	Nervousness, anxiety, or tension
2	GAD 2	Unable to stop or control worrying
3	GAD 3	Excessive worry about various matters
4	GAD 4	Difficulty relaxing
5	GAD 6	Easily annoyed or irritable
Conduct disorders		
6	SDQ 5	Easily getting angry/often losing control
7	SDQ 7	Usually doing what one is toldR
Depressive disorders		
8	PHQ 1	Feeling down, depressed, or hopeless
9	PHQ 2	Little interest or pleasure in doing things
10	PHQ 3	Trouble sleeping
11	PHQ 4	Poor appetite, weight loss, or overeating
12	PHQ 5	Feeling tired or having little energy
13	PHQ 6	Feeling bad about oneself
14	PHQ 7	Trouble concentrating on things
Eating disorders		
15	CHEQ 4	Feeling fat
16	CHEQ 5	Strong desire to lose weight
17	CHEQ 7	Unhappy with one's weight
18	CHEQ 8	Feeling ashamed when seeing one's body
Obsessive‐compulsive disorder		
19	SOCS 1	Feeling compelled to check things, touch or count
20	SOCS 2	Being particularly fussy about keeping hands clean
21	SOCS 3	Repeating things until they seem just right
22	SOCS 4	Difficulties completing schoolwork or home duties because they must be gone over again
23	SOCS 5	Worried about not having done something as desired
Psychotic disorders		
24	CAPE 2	Feeling that some people are not what they seem
Bipolar disorder		
25	ASRM 1	Feeling happier or more joyful than usual
26	ASRM 2	Feeling more self‐confident than usual
27	ASRM 3	Needing less sleep than usual
28	ASRM 4	Talking more than usual
29	ASRM 5	Being more active than usual

*Note*: Each item represents a node of the estimated networks. R: Items reversed in the analyses.

Abbreviations: ASRM, *Altman Self‐Rating Mania Scale* (Altman et al., 1997); CHEQ, *Child Eating Disorder Examination‐Questionnaire* (Kliem et al., 2017); GAD, *Generalized Anxiety Disorder Screener* (Löwe et al., 2007; Spitzer et al., 2006); PHQ, *Patient Health Questionnaire‐9 for Adolescents* (Johnson et al., 2002); SDQ‐S, *Strength and Difficulties Questionnaire*, *Self‐report* (Goodman, 2001); SOCS, *Short Obsessive‐Compulsive Disorder Screener* (Piqueras et al., 2015).

Panel Graphical Vector Autoregression (Panel GVAR) models (Epskamp, 2020) were estimated via the *psychonetrics* package (Epskamp, 2021) to analyze symptom associations in the network. Panel GVAR allows the estimation of two distinct types of network structures: Contemporaneous and temporal networks. Contemporaneous networks represent the average within‐time, within‐person associations between variables after accounting for lagged (temporal) effect and all other variables in the same measurement window (Epskamp, 2020). Temporal networks represent the directed, average within‐person relationships between variables across time, capturing the predictive influence of one variable on other variables (i.e., cross‐lagged effects), on itself (i.e., autoregressions), or as part of feedback loops (i.e., self‐reinforcing cycles where symptoms perpetuate each other over time) (Freichel, Veer, et al., 2023).

As GVAR models assume stationarity, the data were detrended for linear and quadratic effects of time and standardized across waves (Freichel et al., 2024; Freichel, Pfirrmann, et al., 2023b). To fit the network model, a saturated model including all potential edges was first estimated using Full‐Information Maximum Likelihood (FIML) to account for missing data. Full‐Information Maximum Likelihood is a gold‐standard approach (Enders, 2001) that generates unbiased estimates comparable to those obtained through multiple imputation techniques, if the data are missing at random (MAR; Schafer & Graham, 2002). To assess patterns of missingness, comprehensive analyses were conducted, including visualizations of missing data patterns using the naniar package (Tierney & Cook, 2023). Logistic regression models were used to examine whether missingness was related to gender, age or any other model variable (Enders, 2023; Schafer & Graham, 2002). In a second step, standard pruning procedures were applied using a step‐up model search (*α* = 0.05) to eliminate all non‐significant edges from the saturated model and subsequently re‐estimate the model with these edges fixed to zero (Blanken et al., 2022). To visualize the final model, the *qgraph* package was used (Epskamp et al., 2012). Model fit was assessed using standard criteria of good model fit (Kline, 2023; Sivo et al., 2006).

Strength centrality measures were used to assess the relative importance of each node in the network. In the contemporaneous network, node Expected Influence (EI; the sum of all raw edge weights that are connected to each node, representing the overall connectivity of a node within the network), Bridge Expected Influence (BEI; the sum of all raw edge weights connecting a node to nodes from other domains), and Bridge Betweenness (BB; the extent to which a node acts as a connector between different symptom domains by lying on the shortest paths between them) were analyzed (Jones et al., 2021; Robinaugh et al., 2016). Additionally, in the temporal network, in‐strength (i.e., the sum of all incoming absolute edge weights, reflecting how strongly a node is influenced by others) and out‐strength (i.e., the sum of all outgoing absolute edge weights, indicating how strongly a node influences other nodes) were analyzed. Radar plots were used to visualize both in‐strength and out‐strength (Ebrahimi et al., 2021).

## RESULTS

### Sample characteristics

The age of the 1186 participants at baseline ranged from 11 to 17 years (*M* = 14.2, SD = 1.9). Regarding gender, 670 (56.5%) identified as female, 512 (43.2%) as male, and 4 (0.3%) as other. With respect to nationality, 905 (76.3%) participants had a Swiss nationality, 191 (16.0%) held a Swiss and a foreign nationality, and 91 (7.7%) had one or more foreign nationalities. Regarding educational levels, 304 participants (25.6%) were attending primary education, 481 (40.7%) lower secondary education, and 388 (32.6%) upper secondary education. Additionally, 13 (1.1%) participants reported being in other educational programs (e.g., internship, private school, or home‐schooling). The descriptive statistics for the investigated variables (see Table 1) across the five time points are displayed in Supporting Information S1: Appendix S1, Table S2.

### Missing data

Out of the initial 1226 participants who gave consent, 40 (3.3%) did not complete any questionnaire and were excluded from the analysis. Among the remaining sample (*n* = 1186), 1169 (98.6%) completed the baseline assessment (t0), 1125 (94.9%) the three‐month follow‐up (t1), 1095 (92.3%) the six‐month follow‐up (t2), 1073 (90.5%) the nine‐month follow‐up (t3), and 1101 (92.8%) the 12‐month follow‐up (t4). Of the 1186 participants, 1006 (84.8%) provided complete data across all five assessment points (see Supporting Information S1: Appendix S2, Tables S3/S4).

### Estimation of the contemporaneous and temporal model

The saturated network model provided acceptable fit to the data (BIC = 322,541.17, RMSEA = 0.02, CFI = 0.93, TLI = 0.91). The pruned model showed a slightly worse fit (BIC = 317,721.86, RMSEA = 0.02, CFI = 0.84, TLI = 0.84).

### Contemporaneous associations

**FIGURE 1:**
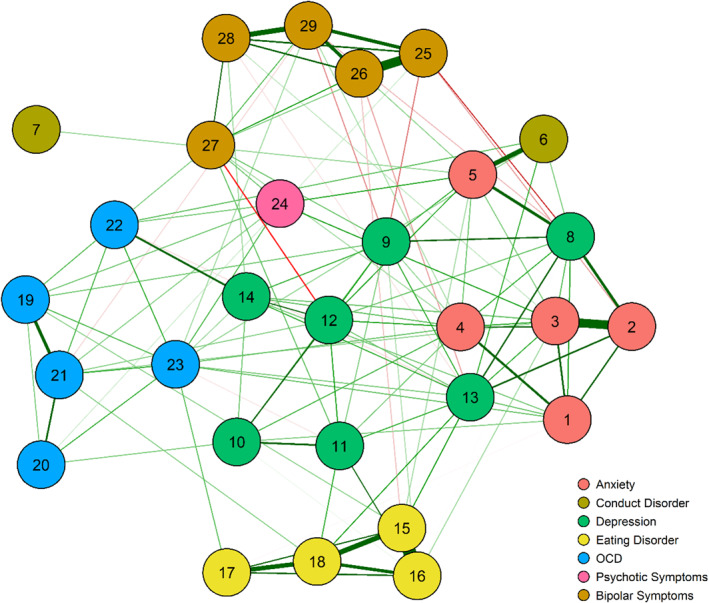
Pruned Contemporaneous Network, showing all significant associations between variables within the same assessment point, while controlling for all other nodes in the network and controlling for temporal effects. Edge thickness reflects association strength, and colors indicate the direction of the association (positive = green; negative = red). Variable names and item descriptions for each node are provided in Table 1.

Figure 1 displays the contemporaneous network. Exact edge weights are provided in Supporting Information S1: Appendix S3, Table S5. Nodes belonging to the same mental disorder domain were more strongly interrelated than those belonging to different mental disorders. In the following part, we report the nodes that ranked above the 75th percentile in the respective centrality index, while Supporting Information S1: Figure S1, Appendix S3 shows the EI values of all nodes in descending order. The nodes with the highest EI in the network were nodes 3 (“Excessive worry about various matters”, EI = 1.08), 15 (“Feeling fat”, EI = 1.03), 16 (“Strong desire to lose weight”, EI = 1.01), 13 (“Feeling bad about oneself”, EI = 1.01), 4 (“Difficulty relaxing”, EI = 0.99), 5 (“Easily annoyed or irritable”, EI = 0.91), and 2 (“Unable to stop or control worrying”, EI = 0.91). The nodes with the highest BEI were nodes 5 (“Easily annoyed or irritable”, BEI = 0.80), 4 (“Difficulty relaxing”, BEI = 0.58), 13 (“Feeling bad about oneself”, BEI = 0.57), 8 (“Feeling down, depressed, or hopeless”, BEI = 0.53), 24 (“Feeling that some people are not what they seem”, BEI = 0.50), and 6 (“Easily getting angry/Often lose control”, BEI = 0.48). The highest BB values were observed for nodes 5 (“Easily annoyed or irritable”, BB = 0.58), 13 (“Feeling bad about oneself”, BB = 0.50), 27 (“Needing less sleep than usual”, BB = 0.45), 11 (“Poor appetite, weight loss, or overeating”, BB = 0.39), 29 (“Being more active than usual”, BB = 0.31), and 23 (“Worried about not having done something as desired”, BB = 0.27). Supporting Information S1: Figure S2, Appendix S3 shows the z‐standardized BEI and BB values of all nodes in descending order.

### Temporal associations

**FIGURE 2:**
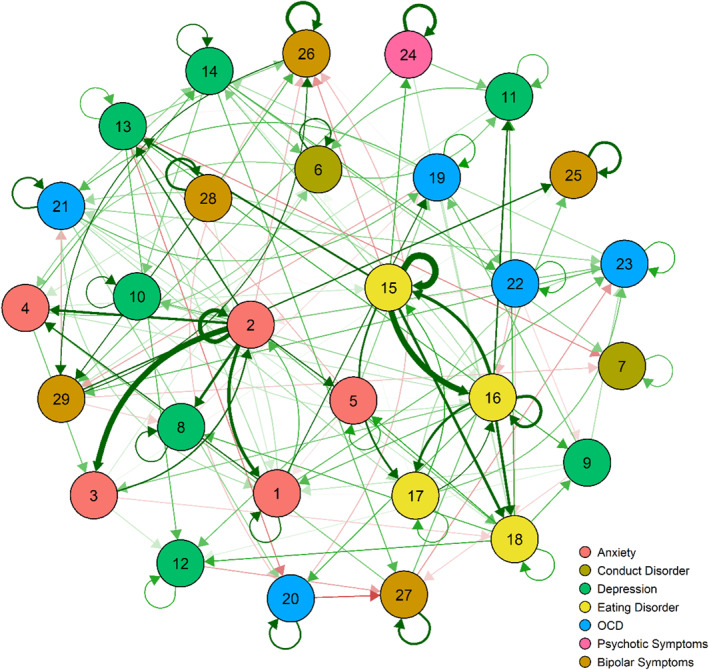
Pruned temporal network showing all directed associations across time while controlling for all other nodes in the network. Edge thickness reflects association strength, and colors indicate direction (positive = green; negative = red). Variable names and item descriptions for each node are provided in Table 1.

Figure 2 displays the temporal network. Node centrality analysis identified node 2 (“Unable to stop or control worrying”) as having the highest out‐strength (*β* = 1.35). Within the anxiety disorder domain, this node exhibited predictive effects on all other nodes (*β* = 0.71) while also showing autoregressive effects (*β* = 0.23). Across domains, this node had the strongest predictive effect on symptoms from the depressive disorder domain (“Feeling down, depressed, or hopeless”: *β* = 0.16; “Feeling bad about oneself”: *β* = 0.12; and “Trouble sleeping”: *β* = 0.06).

The nodes 15 (“Feeling fat”: *β* = 1.09) and 16 (“Strong desire to lose weight”: *β* = 1.04) also exhibited high out‐strength. Both symptoms acted as predictors for all other symptoms within the eating disorder domain (node 15: *β* = 0.57; node 16: *β* = 0.49), exhibited strong autoregressive effects (node 15: *β* = 0.30; node 16: *β* = 0.17), and formed a feedback loop (node 15 to node 16: *β* = 0.28; node 16 to node 15: *β* = 0.18). Between domains, node 15 (“Feeling fat”) showed predictive effects on depressive (e.g., “Feeling bad about oneself”: *β* = 0.13; and “Trouble concentrating on things”: *β* = 0.05), psychotic (“Feeling that some people are not what they seem”: *β* = 0.08), and bipolar symptoms (“Feeling more self‐confident than usual”: *β* = −0.04). Node 16 (“Strong desire to lose weight”) showed predictive effects on anxiety (“Nervousness, anxiety, or tension”: *β* = 0.07, and “Excessive worry about various matters”: *β* = 0.05), depressive (“Poor appetite, weight loss, or overeating”: *β* = 0.12, and “Trouble sleeping”: *β* = 0.04), and OCD symptoms (“Being particularly fussy about keeping hands clean”: *β* = 0.07, and “Repeating things until they seem just right”: *β* = 0.04).

The nodes with the highest in‐strength all belonged to the eating disorder domain (e.g., node 16, “Strong desire to lose weight”: *β* = 0.59; node 15, “Feeling fat”: *β* = 0.58; node 18, “Feeling ashamed when seeing one's body”: *β* = 0.50).

**FIGURE 3:**
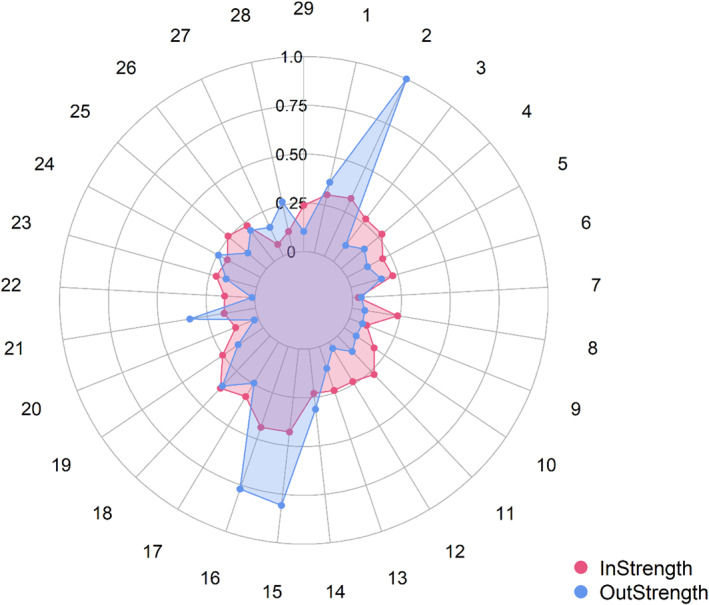
Radar Chart showing the extent to which a node exerts influence on other nodes (out‐strength) and to which extent a node is influenced by other nodes (in‐strength) across time. Values are standardized relative to the maximum observed strength (1.35), with the outermost ring representing the highest observed value scaled to 1. Variable names and item descriptions for each node are provided in Table 1.

The radar chart (Figure 3) illustrates the in‐strength and out‐strength values for the temporal network (see Supporting Information S1: Appendix S4, Tables S6–S8 for exact in‐strength and out‐strength values).

## DISCUSSION

This is the first study to examine contemporaneous and temporal networks of subclinical symptoms across multiple mental disorder domains over 1 year in a large general population sample of adolescents without any current or past diagnosis of a mental disorder.

### Contemporaneous network

Our findings revealed that symptom associations were generally stronger within than across mental disorder domains, consistent with previous research (Boschloo, Van Borkulo, Borsboom, et al., 2016; Wang et al., 2021). Moreover, symptoms from the anxiety (excessive worrying, difficulty relaxing, and irritability), depressive (low self‐esteem), and eating disorder domains (body dissatisfaction and desire to lose weight) were most central in the contemporaneous network. These findings are supported by previous research highlighting the central role of anxiety and depressive symptoms in cross‐sectional psychopathological networks in adolescent general population samples (Black et al., 2022; Contreras et al., 2019; Wang et al., 2021). Further, these results align with staging models of emerging psychopathology, which assume that subclinical stages are characterized by unspecific symptom patterns, particularly anxiety and depressive symptoms (McGorry & Mei, 2021; Shah et al., 2020). The high centrality of eating disorder symptoms, despite their limited associations with other symptom domains, suggests that these symptoms mutually reinforce each other, thereby forming a strongly interconnected community (Smith et al., 2018).

Irritability and low self‐esteem exhibited high values on all three indices including node EI, BB, and BEI. This indicates that they are not only highly interconnected in the network but also function as key bridge symptoms that link different mental disorder domains. This aligns with other network modeling studies (Tseng et al., 2023; Weintraub et al., 2020) and recent meta‐analytic findings identifying irritability —a form of emotional dysregulation linked to deficits in self‐regulatory control processes (Urben et al., 2024) —as a transdiagnostic symptom predicting various mental health problems in adolescents (Chin et al., 2025; Finlay‐Jones et al., 2024). Moreover, low self‐esteem was found to be an important bridge symptom, which is consistent with previous network studies in adolescent and young adult samples showing that low self‐esteem linked symptoms belonging to disordered eating to those of depression and anxiety (Sahlan et al., 2021; Smith et al., 2019). Further, difficulties relaxing, a symptom belonging to the anxiety domain, exhibited high BEI but not BB as it was connected to multiple depressive symptoms, such as feeling depressed, trouble sleeping, and tiredness. This pattern aligns with research showing that difficulty relaxing is closely related to sleep disturbances, which in turn increase the risk for the development of depression (Riemann et al., 2019). In contrast, a reduced need for sleep, which is part of the bipolar disorder domain, exhibited high BB but not BEI as it linked symptoms from multiple disorders, including anxiety, depression, obsessive‐compulsive, psychotic and conduct disorder domains. This is in line with evidence that insufficient sleep, which may be the consequence of a biased perception of one's own sleep need (Short et al., 2013), both results from and contributes to various mental health problems in adolescence (Uccella et al., 2023).

### Temporal network

Uncontrollable worry had the strongest predictive influence on other symptoms over time, suggesting that it may act as a central driver in emerging psychopathology in adolescence. It predicted all other anxiety symptoms and several symptoms across multiple disorder domains, especially within depression symptoms (negative mood, low self‐esteem, and sleep problems). This aligns with previous network (Black et al., 2022; Funkhouser et al., 2021) and meta‐analytic studies identifying worry as a transdiagnostic process in adolescent psychopathology (Egan et al., 2025). Moreover, uncontrollable worry demonstrated high autoregressive effects, suggesting that repetitive and persistent worry can become dysfunctional over time (McLaughlin et al., 2007; Verplanken, 2012) and develop into a mental habit (Verplanken et al., 2007; Watkins, 2008).

The eating disorder symptoms body dissatisfaction (“feeling fat”) and weight concerns (“strong desire to lose weight”) were central in both the contemporaneous and temporal networks. This finding aligns with previous network studies in adolescents and adults with eating disorders (Calugi et al., 2020; Forrest et al., 2019) and is consistent with staging models of eating disorder psychopathology, which characterize early stages by mild restrictive eating behavior and body image disturbances, including body dissatisfaction and weight concerns (Tomba et al., 2024). Body dissatisfaction was predictive of depressive, psychotic, and bipolar symptoms. The association between body dissatisfaction, depressive (low self‐esteem), and bipolar symptoms (lower self‐confidence than usual) aligns with findings that body dissatisfaction in adolescence predicts later depressive episodes and reduced self‐esteem (Bornioli et al., 2021). Additionally, the predictive effect of body dissatisfaction on mistrust (“feeling that some people are not what they seem”) may be explained by negative peer influences, such as appearance‐related teasing and pressure, potentially fostering mistrust in social relationships (Webb & Zimmer‐Gembeck, 2014).

Weight concerns were predictive for anxiety, depressive, and OCD symptoms. This may be explained by the result that excessively thinking about weight and body shape can trigger negative self‐thoughts due to unrealistic societal beauty standards, which may increase stress, depression, and anxiety over time, especially during adolescence (Puccio et al., 2017; Ting et al., 2012). The influence of weight concerns on OCD symptoms supports the hypothesis that the development of eating disorder symptoms might lead to an exacerbation of a pre‐existing predisposition to obsessive‐compulsive symptoms (S. E. Altman & Shankman, 2009). Studies in clinical samples with eating disorders also found a reciprocal relationship between overevaluation of shape and weight control behaviors (Tabri et al., 2015). This may explain the feedback loop we found between “feeling fat” and “strong desire to lose weight” and the strong auto‐regressive effects in our study, which suggests that these two symptoms may form a self‐reinforcing cycle over time.

Notably, depressive symptoms, such as feeling depressed or low self‐esteem, were highly central in the contemporaneous network, but did not act as predictors of other symptoms over time. Instead, they were more influenced by other symptoms than vice versa. This implies that depression in adolescence is more likely to develop as a consequence of other symptoms, such as anxiety or body dissatisfaction, rather than serving as an initial driver of psychopathology (Bornioli et al., 2021; Hankin, 2015).

### Clinical implications

Excessive worry, body dissatisfaction, and weight concerns were central symptoms in both contemporaneous and temporal networks, serving as potential drivers of emerging psychopathology. Irritability and low self‐esteem acted as bridge symptoms, potentially accounting for the emergence of later comorbidities. Early interventions targeting central symptoms may prevent symptom progression. For instance, excessive worry could be effectively targeted through school‐based cognitive‐behavioral therapy (CBT) programs that incorporate cognitive restructuring and exposure‐based strategies (Barrett et al., 2000; Kendall & Hendtke, 2006; Podina et al., 2016). Bridge symptoms may be important transdiagnostic targets as they may reduce the progression of symptoms across mental disorder domains and thereby prevent the development of comorbidity in adolescence (Shah et al., 2020). For example, novel Exposure‐Based CBT approaches seem promising to reduce irritability (Naim et al., 2024).

### Limitations and future research

Several limitations should be acknowledged when interpreting the findings. First, the model fit was not optimal using traditional fit indices. However, no established criteria exist for evaluating fit in network models. In network models, overall fit indices primarily assess the global structure, whereas strong, consistent associations between specific nodes can still offer valuable insights into symptom interactions (Hallquist et al., 2021). Nevertheless, future research should aim to replicate these findings using larger samples and additional time points to validate the robustness and generalizability of the results. Second, subclinical symptoms were assessed every three months over a 12‐month period. While this design allowed the monitoring of short‐term symptom changes, the assessment interval and overall duration may not fully capture the timescales at which symptom dynamics and disorder onset unfold. Rapid symptom fluctuations may occur on shorter timescales, whereas slower developmental processes and low base‐rate transitions (e.g., onset of psychosis or bipolar‐spectrum disorders) may evolve over multiple years (Bringmann et al., 2023; Helmich et al., 2021; Olthof et al., 2023). Furthermore, the broad age range of 11–17 years enabled the investigation of emerging psychopathology across multiple mental disorders but introduced additional heterogeneity in developmental stage and symptom expression, which may complicate interpretation. Future studies could combine intensive longitudinal designs with longer follow‐up periods and consider narrower age bands to better capture both short‐term and long‐term symptom trajectories. Third, neurodevelopmental traits were not assessed in this study. While this allowed us to focus on emerging psychopathology in adolescents without pre‐existing mental disorders, it limits our understanding of how neurodevelopmental traits, which typically have their onset in childhood, might influence symptom development. Future research should therefore examine their potential role in emerging psychopathology, given possible shared transdiagnostic mechanisms (Cervin, 2023; Sonuga‐Barke & Halperin, 2010). Lastly, this study relied on self‐report questionnaires to assess psychopathology, but such measures are subject to potential recall and social desirability biases (Bone et al., 2021; van de Mortel, 2008). Future research should therefore integrate multi‐informant data (e.g., parent or clinician reports) and objective behavioral measures (e.g., digital phenotyping data) to enhance the robustness of symptom assessments (Edgar et al., 2009). Despite these limitations, this study has several strengths. The exclusion of clinically relevant cases and the inclusion of a broad range of symptoms from various disorder domains provide valuable insights into the transdiagnostic network structure of emerging psychopathology in adolescence. Moreover, the use of 3‐month time intervals over 1 year combined with novel statistical methods may improve the detection of transient and complex symptom interactions.

## CONCLUSION

The EMERGE‐study aimed to better understand the cross‐sectional and temporal transdiagnostic network structure of emerging psychopathology in adolescence. In the cross‐sectional network, symptoms belonging to depression and anxiety were the most strongly connected to other symptoms and acted as important links between different disorder domains. In the temporal network, symptoms belonging to anxiety and eating disorders were particularly influential in predicting other symptoms over time. These findings underscore the pivotal role of these symptoms in the development and maintenance of subclinical psychopathology. By identifying and targeting the most central and interconnected symptoms, this knowledge can enhance early detection and prevention strategies, ultimately supporting individuals who may need care but are not yet seeking help.

## AUTHOR CONTRIBUTIONS

**Xenia A. Häfeli**: Conceptualization (supporting); data curation (lead); project administration (lead); writing—original draft preparation (lead); writing—review and editing (equal). **Larisa Morosan**: Formal analysis (supporting); writing—review and editing (equal). **Anja Hirsig**: Investigation (lead); writing—review and editing (equal). **Stefanie J. Schmidt**: Conceptualization (lead); funding acquisition (lead); methodology (lead); supervision (lead); writing—review and editing (equal).

## CONFLICT OF INTEREST STATEMENT

The authors declare no conflicts of interest.

## ETHICAL CONSIDERATIONS

Informed consent was obtained from all participants aged 14 years and older. For participants younger than 14, informed consent was obtained from a parent or legal guardian, and assent was obtained from the minor. Consent was provided electronically in both oral and written form. The authors assert that all procedures contributing to this work comply with the ethical standards of the relevant national and institutional committees on human experimentation and with the Helsinki Declaration of 1975, as revised in 2013. All procedures involving human subjects/patients were approved by the Cantonal Ethics Committee (KEK) Bern (ID 2020‐02108) on 28 June 2021.

## Supporting information

Supporting Information S1

## Data Availability

The data that support the findings of this study are available from the PI, SJS, upon reasonable request.
