# Supplementary material for: Transdiagnostic symptom networks in adolescent psychopathology: A longitudinal panel network analysis
Source: JCPP Adv. 2026 Apr 6:e70118. Online ahead of print. doi: 10.1002/jcv2.70118 (PMC13339512; doi:10.1002/jcv2.70118)
Supplement: Supplementary file 1 — Supporting Information S1 [file JCV2-9999-e70118-s001.docx]

# **Transdiagnostic Symptom Networks in Adolescent Psychopathology:**

# **A Longitudinal Panel Network Analysis**

**Supporting Information**

**Appendix S1: Descriptive Analysis**

**Table S1** *Originally assessed Symptoms in the EMERGE-study*

| Node | Variable | Item Description |  |  |  |  |
| --- | --- | --- | --- | --- | --- | --- |
|  |  | Bipolar Disorder | Mean (SD), range |  |  |  |
| 3 | ASRM 1 | Feeling happier or more joyful than usual | 1.63 (1.16), 0–4 |  |  |  |
| 4 | ASRM 2 | Feeling more self-confident than usual | 1.52 (1.17), 0–4 |  |  |  |
| 5 | ASRM 3 | Needing less sleep than usual | 0.72 (1.02), 0–4 |  |  |  |
| 6 | ASRM 4 | Talking more than usual | 0.88 (0.98), 0–4 |  |  |  |
| 7 | ASRM 5 | Being more active than usual | 1.19 (1.12), 0–4 |  |  |  |
|  |  | Anxiety Disorder | Mean (SD), range |  |  |  |
| 8 | GAD 1 | Nervousness, anxiety, or tension | 0.79 (0.63), 0–3 |  |  |  |
| 9 | GAD 2 | Unable to stop or control worrying | 0.52 (0.69), 0–3 |  |  |  |
| 10 | GAD 3 | Excessive worry about various matters | 0.63 (0.72), 0–3 |  |  |  |
| 11 | GAD 4 | Difficulty relaxing | 0.63 (0.73), 0–3 |  |  |  |
| 12 | GAD 5 | Restlessness that makes it hard to sit still | 0.40 (0.63), 0–3 |  |  |  |
| 13 | GAD 6 | Easily annoyed or irritable | 0.75 (0.75), 0–3 |  |  |  |
| 14 | GAD 7 | Feeling afraid of something awful happening | 0.34 (0.60), 0–3 |  |  |  |
|  |  | Depressive Disorder | Mean (SD), range |  |  |  |
| 15 | PHQ 1 | Feeling down, depressed, or hopeless | 0.56 (0.68), 0–3 |  |  |  |
| 16 | PHQ 2 | Little interest or pleasure in doing things. | 0.55 (0.66), 0–3 |  |  |  |
| 17 | PHQ 3 | Trouble sleeping | 0.70 (0.85), 0–3 |  |  |  |
| 18 | PHQ 4 | Poor appetite, weight loss, or overeating | 0.28 (0.59), 0–3 |  |  |  |
| 19 | PHQ 5 | Feeling tired or having little energy | 0.88 (0.76), 0–3 |  |  |  |
| 20 | PHQ 6 | Feeling bad about oneself | 0.47 (0.71), 0–3 |  |  |  |
| 21 | PHQ 7 | Trouble concentrating on things | 0.51 (0.69), 0–3 |  |  |  |
| 22 | PHQ 8 | Psychomotor agitation or retardation | 0.16 (0.41), 0–3 |  |  |  |
| 23 | PHQ 9 | Suicidal ideation | 0.11 (0.36), 0–3 |  |  |  |
|  |  | Somatic Symptoms | Mean (SD), range |  |  |  |
| 24 | SSS 1 | Stomach or bowel problems | 0.70 (0.89), 0–4 |  |  |  |
| 25 | SSS 2 | Back pain | 0.76 (0.94), 0–4 |  |  |  |
| 26 | SSS 3 | Pain in arms, legs, or joints | 0.74 (0.86), 0–4 |  |  |  |
| 27 | SSS 4 | Headaches | 0.93 (1.01), 0–4 |  |  |  |
| 28 | SSS 5 | Chest pain or shortness of breath | 0.30 (0.68), 0–4 |  |  |  |
| 29 | SSS 6 | Dizziness | 0.57 (0.88), 0–4 |  |  |  |
|  |  | Substance Abuse | Proportion (%), range |  |  |  |
| 30 | CRFT 1 | Driven under the influence of substances, or ridden with someone who had consumed substances | Yes (1): 5.63%, 0–1 |  |  |  |
| 31 | CRFT 2 | Consumed substances to relax, feel better, or to feel more comfortable around friends or acquaintances | Yes (1): 4.70%, 0–1 |  |  |  |
| 32 | CRFT 3 | Consumed substances when alone | Yes (1): 1.93%, 0–1 |  |  |  |
| 33 | CRFT 4 | Received advice from family/friends to consume less | Yes (1): 2.51%, 0–1 |  |  |  |
| 34 | CRFT 5 | Forgotten something done while consuming substances | Yes (1): 9.06%, 0–1 |  |  |  |
| 35 | CRFT 6 | Had trouble with others when consuming substances | Yes (1): 2.18%, 0–1 |  |  |  |
|  |  | Eating Disorder | Mean (SD), range |  | |  |
| 36 | CHEQ 1 | Eating less to change body shape or weight | 0.63 (1.26), 0–6 |  |  |  |
| 37 | CHEQ 2 | Avoiding food you like to change figure or weight | 0.46 (1.13), 0–6 |  |  |  |
| 38 | CHEQ 3 | Difficulties concentrating on things that interest you because of thinking about food or calories | 0.17 (0.64), 0–6 |  |  |  |
| 39 | CHEQ 4 | Feeling fat | 0.83 (1.48), 0–6 |  |  |  |
| 40 | CHEQ 5 | Strong desire to lose weight | 0.68 (1.49), 0–6 |  |  |  |
| 41 | CHEQ 6 | Feeling guilty after eating | 0.48 (0.99), 0–6 |  |  |  |
| 42 | CHEQ 7 | Unhappy with one's weight | 1.21 (1.64), 0–6 |  |  |  |
| 43 | CHEQ 8 | Feeling ashamed when seeing one's body | 0.79 (1.27), 0–6 |  |  |  |
|  |  | Psychotic Disorder | Mean (SD), range |  |  |  |
| 44 | CAPE 1 | Feeling that people are making hidden insinuations about oneself or saying things with double meanings | 0.45 (0.90), 0–9 |  |  |  |
| 45 | CAPE 2 | Feeling that some people are not what they seem | 0.71 (1.16), 0–9 |  |  |  |
| 46 | CAPE 3 | Feeling somehow followed | 0.29 (0.81), 0–9 |  |  |  |
| 47 | CAPE 4 | Feeling that a plot is being concocted against oneself | 0.16 (0.63), 0–9 |  |  |  |
| 48 | CAPE 5 | Feeling stared at because of one's appearance | 0.46 (1.12), 0–9 |  |  |  |
| 49 | CAPE 6 | Feeling that electronic devices influencing thoughts | 0.27 (0.80), 0–9 |  |  |  |
| 50 | CAPE 7 | Feeling that thoughts are being pulled out of the head | 0.10 (0.44), 0–6 |  |  |  |
| 51 | CAPE 8 | Feeling that certain thoughts are being inserted | 0.13 (0.44), 0–4 |  |  |  |
| 52 | CAPE 9 | Thoughts so vivid it's feared others can hear them | 0.15 (0.57), 0–6 |  |  |  |
| 53 | CAPE 10 | Thoughts repeated (like an echo) | 0.06 (0.34), 0–6 |  |  |  |
| 54 | CAPE 11 | Feeling under the control of some force or power | 0.07 (0.39), 0–4 |  |  |  |
| 55 | CAPE 12 | Hearing voices | 0.14 (0.61), 0–6 |  |  |  |
| 56 | CAPE 13 | Hearing voices that talk to each other | 0.05 (0.35), 0–6 |  |  |  |
| 57 | CAPE 14 | Feeling that a double has taken the place of a relative, friend, or acquaintance | 0.06 (0.25), 0–9 |  |  |  |
| 58 | CAPE 15 | Seeing things, people, or animals that others can't see | 0.06 (0.34), 0–4 |  |  |  |
|  |  | Obsessive-Compulsive Disorder | Mean (SD), range |  |  |  |
| 59 | SOCS 1 | Feeling compelled to check things, touch or count | 0.37 (0.56), 0–2 |  |  |  |
| 60 | SOCS 2 | Being particularly fussy about keeping hands clean | 0.34 (0.57), 0–2 |  |  |  |
| 61 | SOCS 3 | Repeating things until they seem just right | 0.43 (0.60), 0–2 |  |  |  |
| 62 | SOCS 4 | Difficulties completing schoolwork or home duties because they must be gone over again | 0.29 (0.51), 0–2 |  |  |  |
| 63 | SOCS 5 | Worried about not having done something as desired | 0.67 (0.65), 0–2 |  |  |  |
|  |  | Conduct Disorder | Mean (SD), range |  |  |  |
| 64 | SDQ 1 | Easily getting angry/Often lose control | 0.36 (0.54), 0–2 |  |  |  |
| 65 | SDQ 2 | Usually doing what one is told^R^ | 0.68 (0.59), 0–2 |  |  |  |
| 66 | SDQ 3 | Often fighting; forcing others to things | 0.08 (0.29), 0–2 |  |  |  |
| 67 | SDQ 4 | Being claimed to often lie or cheat | 0.17 (0.41), 0–2 |  |  |  |
| 68 | SDQ 5 | Taking things that don't belong to you | 0.10 (0.32), 0–2 |  |  |  |
| *Note*. ASRM: *Altman Self-Rating Mania* Scale (ASRM; 37). CAPE: *Community Assessment of Psychic Experiences – Positive* (CAPE-P15; 47). CHEQ*: Child Eating Disorder Examination-Questionnaire* (CHEDE-Q8; 44). CRFT: *Car, Relax, Alone, Forget, Family/Friends, and Trouble scale* (CRAFT-d; 52). GAD: *Generalized Anxiety Disorder Screener* (GAD-7; 33,34). PHQ: *Patient Health Questionnaire-9 for Adolescents* (PHQ-A; 42). SDQ: *Strength and Difficulties Questionnaire* (SDQ; 40). SOCS: *Short Obsessive-Compulsive Disorder Screener* (SOCS; 45). SSS: *Somatic Symptom Scale* (SSS-8; 50). Each item represents a node of the estimated networks. The table also provides the means and the range of each item. R: Items reversed in the analyses. | | | |  |  |  |

**Table S2** *Descriptive statistics for each variable at all test-timepoints*

|  | **t0** | |  | | **t1** | |  | | **t2** | |  | | **t3** | |  | | **t4** | |  | |
| --- | --- | --- | --- | --- | --- | --- | --- | --- | --- | --- | --- | --- | --- | --- | --- | --- | --- | --- | --- | --- |
|  | **Mean** | **(SD)** | | **Range** | **Mean** | **(SD)** | | **Range** | **Mean** | **(SD)** | | **Range** | **Mean** | **(SD)** | | **Range** | **Mean** | **(SD)** | | **Range** |
| GAD 1 | 0.79 | 0.62 | | 0-3 | 0.88 | 0.66 | | 0-3 | 0.87 | 0.68 | | 0-3 | 0.88 | 0.69 | | 0-3 | 0.81 | 0.70 | | 0-3 |
| GAD 2 | 0.51 | 0.69 | | 0-3 | 0.57 | 0.71 | | 0-3 | 0.63 | 0.74 | | 0-3 | 0.61 | 0.72 | | 0-3 | 0.55 | 0.73 | | 0-3 |
| GAD 3 | 0.63 | 0.72 | | 0-3 | 0.70 | 0.72 | | 0-3 | 0.69 | 0.74 | | 0-3 | 0.72 | 0.77 | | 0-3 | 0.65 | 0.77 | | 0-3 |
| GAD 4 | 0.62 | 0.73 | | 0-3 | 0.73 | 0.75 | | 0-3 | 0.73 | 0.77 | | 0-3 | 0.76 | 0.80 | | 0-3 | 0.67 | 0.76 | | 0-3 |
| GAD 6 | 0.74 | 0.74 | | 0-3 | 0.89 | 0.74 | | 0-3 | 0.85 | 0.75 | | 0-3 | 0.84 | 0.76 | | 0-3 | 0.68 | 0.74 | | 0-3 |
| SDQ 5 | 0.36 | 0.54 | | 0-2 | 0.45 | 0.60 | | 0-2 | 0.45 | 0.61 | | 0-2 | 0.45 | 0.61 | | 0-2 | 0.37 | 0.57 | | 0-2 |
| SDQ 7 | 0.68 | 0.59 | | 0-2 | 0.64 | 0.60 | | 0-2 | 0.65 | 0.60 | | 0-2 | 0.65 | 0.62 | | 0-2 | 0.60 | 0.61 | | 0-2 |
| PHQ 1 | 0.56 | 0.67 | | 0-3 | 0.64 | 0.72 | | 0-3 | 0.65 | 0.72 | | 0-3 | 0.65 | 0.73 | | 0-3 | 0.60 | 0.72 | | 0-3 |
| PHQ 2 | 0.54 | 0.66 | | 0-3 | 0.63 | 0.71 | | 0-3 | 0.65 | 0.75 | | 0-3 | 0.61 | 0.70 | | 0-3 | 0.59 | 0.68 | | 0-3 |
| PHQ 3 | 0.70 | 0.86 | | 0-3 | 0.75 | 0.85 | | 0-3 | 0.78 | 0.87 | | 0-3 | 0.76 | 0.85 | | 0-3 | 0.70 | 0.85 | | 0-3 |
| PHQ 4 | 0.28 | 0.59 | | 0-3 | 0.37 | 0.67 | | 0-3 | 0.42 | 0.72 | | 0-3 | 0.42 | 0.71 | | 0-3 | 0.39 | 0.71 | | 0-3 |
| PHQ 5 | 0.88 | 0.76 | | 0-3 | 0.97 | 0.83 | | 0-3 | 0.96 | 0.84 | | 0-3 | 0.97 | 0.82 | | 0-3 | 0.93 | 0.83 | | 0-3 |
| PHQ 6 | 0.47 | 0.70 | | 0-3 | 0.56 | 0.77 | | 0-3 | 0.53 | 0.79 | | 0-3 | 0.56 | 0.78 | | 0-3 | 0.51 | 0.75 | | 0-3 |
| PHQ 7 | 0.51 | 0.69 | | 0-3 | 0.64 | 0.75 | | 0-3 | 0.62 | 0.77 | | 0-3 | 0.61 | 0.75 | | 0-3 | 0.56 | 0.74 | | 0-3 |
| CHEQ 4 | 0.83 | 1.48 | | 0-6 | 0.94 | 1.59 | | 0-6 | 0.97 | 1.59 | | 0-6 | 0.99 | 1.58 | | 0-6 | 0.97 | 1.58 | | 0-6 |
| CHEQ 5 | 0.68 | 1.49 | | 0-6 | 0.75 | 1.55 | | 0-6 | 0.80 | 1.59 | | 0-6 | 0.81 | 1.59 | | 0-6 | 0.78 | 1.56 | | 0-6 |
| CHEQ 7 | 1.21 | 1.64 | | 0-6 | 1.34 | 1.71 | | 0-6 | 1.33 | 1.66 | | 0-6 | 1.39 | 1.69 | | 0-6 | 1.41 | 1.73 | | 0-6 |
| CHEQ 8 | 0.79 | 1.28 | | 0-6 | 0.93 | 1.44 | | 0-6 | 0.94 | 1.40 | | 0-6 | 0.99 | 1.46 | | 0-6 | 0.96 | 1.43 | | 0-6 |
| SOCS 1 | 0.37 | 0.56 | | 0-2 | 0.42 | 0.59 | | 0-2 | 0.44 | 0.60 | | 0-2 | 0.42 | 0.60 | | 0-2 | 0.37 | 0.57 | | 0-2 |
| SOCS 2 | 0.34 | 0.56 | | 0-2 | 0.37 | 0.59 | | 0-2 | 0.34 | 0.58 | | 0-2 | 0.36 | 0.58 | | 0-2 | 0.33 | 0.57 | | 0-2 |
| SOCS 3 | 0.42 | 0.59 | | 0-2 | 0.47 | 0.61 | | 0-2 | 0.48 | 0.61 | | 0-2 | 0.46 | 0.62 | | 0-2 | 0.41 | 0.59 | | 0-2 |
| SOCS 4 | 0.28 | 0.51 | | 0-2 | 0.29 | 0.54 | | 0-2 | 0.29 | 0.53 | | 0-2 | 0.28 | 0.52 | | 0-2 | 0.24 | 0.48 | | 0-2 |
| SOCS 5 | 0.67 | 0.65 | | 0-2 | 0.68 | 0.64 | | 0-2 | 0.69 | 0.66 | | 0-2 | 0.69 | 0.66 | | 0-2 | 0.63 | 0.65 | | 0-2 |
| CAPE 2 | 0.69 | 1.14 | | 0-9 | 0.80 | 1.32 | | 0-9 | 0.79 | 1.31 | | 0-9 | 0.75 | 1.35 | | 0-9 | 0.67 | 1.23 | | 0-9 |
| ASRM 1 | 1.63 | 1.16 | | 0-4 | 1.54 | 1.14 | | 0-4 | 1.37 | 1.14 | | 0-4 | 1.30 | 1.12 | | 0-4 | 1.16 | 1.11 | | 0-4 |
| ASRM 2 | 1.52 | 1.17 | | 0-4 | 1.43 | 1.16 | | 0-4 | 1.32 | 1.13 | | 0-4 | 1.27 | 1.14 | | 0-4 | 1.07 | 1.10 | | 0-4 |
| ASRM 3 | 0.72 | 1.01 | | 0-4 | 0.72 | 0.97 | | 0-4 | 0.68 | 0.99 | | 0-4 | 0.65 | 0.94 | | 0-4 | 0.60 | 0.93 | | 0-4 |
| ASRM 4 | 0.88 | 0.98 | | 0-4 | 0.83 | 0.96 | | 0-4 | 0.78 | 0.92 | | 0-4 | 0.72 | 0.87 | | 0-4 | 0.64 | 0.84 | | 0-4 |
| ASRM 5 | 1.19 | 1.12 | | 0-4 | 1.13 | 1.10 | | 0-4 | 1.05 | 1.05 | | 0-4 | 1.01 | 1.02 | | 0-4 | 0.90 | 1.02 | | 0-4 |

*Note.* ASRM: Altman Self-Rating Mania Scale (ASRM: Altman et al., 1997). CHEQ*: Child Eating Disorder Examination-Questionnaire* (CHEDE-Q8; Kliem et al., 2017). GAD: *Generalized Anxiety Disorder Screener* (GAD-7; Löwe et al., 2007; Spitzer et al., 2006). PHQ: *Patient Health Questionnaire-9 for Adolescents* (PHQ-A; Johnson et al., 2002). SDQ: *Strength and Difficulties Questionnaire* (SDQ; Goodman, 2001). SOCS: *Short Obsessive-Compulsive Disorder Screener* (SOCS; Piqueras et al., 2015).

**Appendix S2: Missingness Analysis**

**Table S3** *Response completeness for each questionnaire at each assessment point*

| Questionnaire | Empty |  | Partial |  | Complete |  |
| --- | --- | --- | --- | --- | --- | --- |
|  | n | % | n | % | n | % |
| t0 | 12 | 1.0 | 5 | 0.4 | 1169 | 98.6 |
| t1 | 52 | 4.4 | 9 | 0.8 | 1125 | 94.9 |
| t2 | 75 | 6.3 | 16 | 1.3 | 1095 | 92.3 |
| t3 | 103 | 8.7 | 10 | 0.8 | 1073 | 90.5 |
| t4 | 79 | 6.7 | 6 | 0.5 | 1101 | 92.8 |
| mean | 64.2 | 5.4 | 9.2 | 0.8 | 1112.6 | 93.8 |
| t0-t4 |  |  |  |  | 1006 | 84.8 |

*Note.* N = 1186. Empty = no items answered; Partial = some items answered; Complete = all items answered. The t0–t4 row indicates participants who completed all five assessment points.

**Table S4** *Percentages of missing values for each variable at each assessment point*

| **Node** | **Measure** | **t0** | **t1** | **t2** | **t3** | **t4** |
| --- | --- | --- | --- | --- | --- | --- |
| 1 | GAD 1 | 1.26 | 4.47 | 6.83 | 8.77 | 6.91 |
| 2 | GAD 2 | 1.26 | 4.55 | 6.83 | 8.77 | 6.91 |
| 3 | GAD 3 | 1.26 | 4.55 | 6.83 | 8.77 | 6.91 |
| 4 | GAD 4 | 1.26 | 4.55 | 6.83 | 8.77 | 6.91 |
| 5 | GAD 6 | 1.26 | 4.55 | 6.83 | 8.77 | 6.91 |
| 6 | SDQ 5 | 1.43 | 5.14 | 7.50 | 9.53 | 7.08 |
| 7 | SDQ 7 | 1.43 | 5.14 | 7.50 | 9.53 | 7.08 |
| 8 | PHQ 1 | 1.43 | 4.64 | 7.00 | 9.02 | 7.00 |
| 9 | PHQ 2 | 1.43 | 4.64 | 7.00 | 9.02 | 7.00 |
| 10 | PHQ 3 | 1.43 | 4.64 | 7.00 | 9.02 | 7.00 |
| 11 | PHQ 4 | 1.43 | 4.64 | 7.00 | 9.02 | 7.00 |
| 12 | PHQ 5 | 1.43 | 4.64 | 7.00 | 9.02 | 7.00 |
| 13 | PHQ 6 | 1.43 | 4.64 | 7.00 | 9.02 | 7.00 |
| 14 | PHQ 7 | 1.43 | 4.64 | 7.00 | 9.02 | 7.00 |
| 15 | CHEQ 4 | 1.43 | 4.97 | 6.91 | 9.11 | 7.00 |
| 16 | CHEQ 5 | 1.43 | 4.97 | 6.91 | 9.11 | 7.00 |
| 17 | CHEQ 7 | 1.43 | 4.97 | 6.91 | 9.11 | 7.00 |
| 18 | CHEQ 8 | 1.43 | 4.97 | 6.91 | 9.11 | 7.00 |
| 19 | SOCS 1 | 1.43 | 5.14 | 7.50 | 9.36 | 7.08 |
| 20 | SOCS 2 | 1.43 | 5.14 | 7.50 | 9.36 | 7.08 |
| 21 | SOCS 3 | 1.43 | 5.14 | 7.50 | 9.36 | 7.08 |
| 22 | SOCS 4 | 1.43 | 5.14 | 7.50 | 9.36 | 7.08 |
| 23 | SOCS 5 | 1.43 | 5.14 | 7.50 | 9.36 | 7.08 |
| 24 | CAPE 2 | 1.43 | 4.97 | 7.08 | 9.19 | 7.00 |
| 25 | ASRM 1 | 1.01 | 4.38 | 6.41 | 8.68 | 6.75 |
| 26 | ASRM 2 | 1.01 | 4.38 | 6.41 | 8.68 | 6.75 |
| 27 | ASRM 3 | 1.01 | 4.38 | 6.41 | 8.68 | 6.75 |
| 28 | ASRM 4 | 1.01 | 4.38 | 6.41 | 8.68 | 6.75 |
| 29 | ASRM 5 | 1.01 | 4.38 | 6.41 | 8.68 | 6.75 |

*Note.* ASRM: Altman Self-Rating Mania Scale (ASRM: Altman et al., 1997). CHEQ*: Child Eating Disorder Examination-Questionnaire* (CHEDE-Q8; Kliem et al., 2017). GAD: *Generalized Anxiety Disorder Screener* (GAD-7; Löwe et al., 2007; Spitzer et al., 2006). PHQ: *Patient Health Questionnaire-9 for Adolescents* (PHQ-A; Johnson et al., 2002). SDQ: *Strength and Difficulties Questionnaire* (SDQ; Goodman, 2001). SOCS: *Short Obsessive-Compulsive Disorder Screener* (SOCS; Piqueras et al., 2015).

**Appendix S3: Contemporaneous Network Analysis**

**Table S5** *Exact edge weights for the contemporaneous network.*

| **Node** | **1** | **2** | **3** | **4** | **5** | **6** | **7** | **8** | **9** | **10** | **11** | **12** | **13** | **14** | **15** |
| --- | --- | --- | --- | --- | --- | --- | --- | --- | --- | --- | --- | --- | --- | --- | --- |
| **1** |  |  |  |  |  |  |  |  |  |  |  |  |  |  |  |
| **2** | 0.13 |  |  |  |  |  |  |  |  |  |  |  |  |  |  |
| **3** | 0.13 | 0.37 |  |  |  |  |  |  |  |  |  |  |  |  |  |
| **4** | 0.15 | 0.12 | 0.09 |  |  |  |  |  |  |  |  |  |  |  |  |
| **5** | 0 | 0 | 0.05 | 0.06 |  |  |  |  |  |  |  |  |  |  |  |
| **6** | 0 | 0 | 0 | 0 | 0.23 |  |  |  |  |  |  |  |  |  |  |
| **7** | 0 | 0 | 0 | 0 | 0.04 | 0 |  |  |  |  |  |  |  |  |  |
| **8** | 0.11 | 0.17 | 0.07 | 0.09 | 0.17 | 0.05 | 0 |  |  |  |  |  |  |  |  |
| **9** | 0 | 0 | 0 | 0.04 | 0.09 | 0 | 0 | 0.12 |  |  |  |  |  |  |  |
| **10** | 0.05 | 0 | 0 | 0.08 | 0 | 0 | 0 | 0 | 0.08 |  |  |  |  |  |  |
| **11** | 0 | 0 | 0 | 0.06 | 0 | 0 | 0 | 0 | 0.05 | 0.13 |  |  |  |  |  |
| **12** | 0 | 0 | 0 | 0.1 | 0.1 | 0 | 0 | 0.06 | 0.1 | 0.12 | 0.1 |  |  |  |  |
| **13** | 0.06 | 0.13 | 0.09 | 0 | 0 | 0.08 | 0 | 0.12 | 0.09 | 0 | 0.09 | 0.06 |  |  |  |
| **14** | 0.06 | 0.03 | 0.06 | 0.07 | 0 | 0 | 0 | 0 | 0.08 | 0.06 | 0 | 0.11 | 0.09 |  |  |
| **15** | 0 | 0 | 0 | 0 | 0.04 | 0 | 0 | 0 | 0 | 0 | 0 | 0 | 0.11 | 0 |  |
| **16** | 0 | 0 | 0.04 | 0 | 0 | 0 | 0 | 0 | 0.03 | 0.01 | 0.11 | 0 | 0 | 0 | 0.52 |
| **17** | 0 | 0 | 0 | 0 | 0 | 0 | 0 | 0 | 0 | 0 | 0 | 0 | 0 | 0 | 0.11 |
| **18** | 0 | 0 | 0 | 0 | 0 | 0 | 0 | 0 | 0 | 0 | 0.09 | 0 | 0.1 | 0 | 0.24 |
| **19** | 0 | 0 | 0 | 0 | 0 | 0 | 0 | 0 | 0.06 | 0 | 0 | 0 | 0 | 0 | 0.05 |
| **20** | 0 | 0 | 0 | 0 | 0 | 0 | 0 | 0 | 0 | 0.05 | 0 | 0 | 0 | 0 | 0 |
| **21** | 0 | 0 | 0.05 | 0 | 0 | 0 | 0 | 0 | 0 | 0 | 0 | 0.05 | 0 | 0 | 0 |
| **22** | 0 | 0 | 0 | 0 | 0 | 0.07 | 0 | 0 | 0 | 0 | 0 | 0 | -0.01 | 0.14 | 0 |
| **23** | 0.06 | 0 | 0 | 0.06 | 0 | 0.05 | 0 | 0 | 0 | 0 | -0.01 | 0 | 0.06 | 0.04 | 0 |
| **24** | 0 | 0 | 0.1 | 0 | 0.09 | 0 | 0 | 0 | 0 | 0 | 0 | 0 | 0 | 0 | 0 |
| **25** | 0 | -0.04 | 0 | 0 | 0 | 0 | 0 | -0.09 | -0.06 | 0 | 0 | 0 | 0 | 0 | 0 |
| **26** | 0 | 0 | 0 | 0 | 0 | 0 | 0 | 0 | 0 | 0 | 0 | 0 | -0.04 | 0 | -0.03 |
| **27** | 0 | 0 | 0 | 0.04 | 0 | 0 | 0 | 0 | 0.06 | 0 | 0.07 | -0.12 | 0 | 0 | 0 |
| **28** | 0 | 0 | 0.03 | 0 | 0 | 0 | 0 | 0 | 0 | 0 | 0 | 0 | -0.01 | 0.04 | 0 |
| **29** | 0 | 0 | 0 | 0.04 | 0.05 | 0 | 0 | -0.03 | -0.05 | 0 | 0 | 0 | 0 | 0 | 0 |
| **30** | 0 | 0 | 0 | 0 | 0 | 0 | 0 | 0 | 0 | 0 | 0.09 | 0 | 0.1 | 0 | 0.24 |

| **Node** | **16** | **17** | **18** | **19** | **20** | **21** | **22** | **23** | **24** | **25** | **26** | **27** | **28** | **29** |
| --- | --- | --- | --- | --- | --- | --- | --- | --- | --- | --- | --- | --- | --- | --- |
| **16** |  |  |  |  |  |  |  |  |  |  |  |  |  |  |
| **17** | 0.12 |  |  |  |  |  |  |  |  |  |  |  |  |  |
| **18** | 0.18 | 0.22 |  |  |  |  |  |  |  |  |  |  |  |  |
| **19** | 0 | 0 | 0 |  |  |  |  |  |  |  |  |  |  |  |
| **20** | 0 | 0 | 0 | 0.05 |  |  |  |  |  |  |  |  |  |  |
| **21** | 0 | 0 | 0.05 | 0.18 | 0.13 |  |  |  |  |  |  |  |  |  |
| **22** | 0 | 0 | 0 | 0.07 | 0 | 0.08 |  |  |  |  |  |  |  |  |
| **23** | 0 | 0.07 | 0 | 0.08 | 0.08 | 0.09 | 0.09 |  |  |  |  |  |  |  |
| **24** | 0 | 0 | 0 | 0.06 | 0 | 0.05 | 0.06 | 0.08 |  |  |  |  |  |  |
| **25** | 0 | 0 | 0 | 0 | 0.02 | 0 | 0 | 0 | 0 |  |  |  |  |  |
| **26** | 0 | 0 | 0 | 0 | 0 | 0 | 0 | 0 | 0 | 0.39 |  |  |  |  |
| **27** | 0 | 0 | 0 | 0 | 0 | 0 | 0.05 | 0 | 0.06 | 0.05 | 0.1 |  |  |  |
| **28** | 0 | 0 | 0 | 0 | 0 | 0 | 0 | 0 | 0 | 0.13 | 0.13 | 0.11 |  |  |
| **29** | 0 | 0 | 0 | 0 | 0 | -0.02 | 0 | 0.04 | 0 | 0.19 | 0.19 | 0.07 | 0.25 |  |
| **30** | 0 | 0.12 | 0.18 | 0 | 0 | 0 | 0 | 0 | 0 | 0 | 0 | 0 | 0 | 0 |

**Figure S1** *Node Centrality measure for the contemporaneous network.*

*
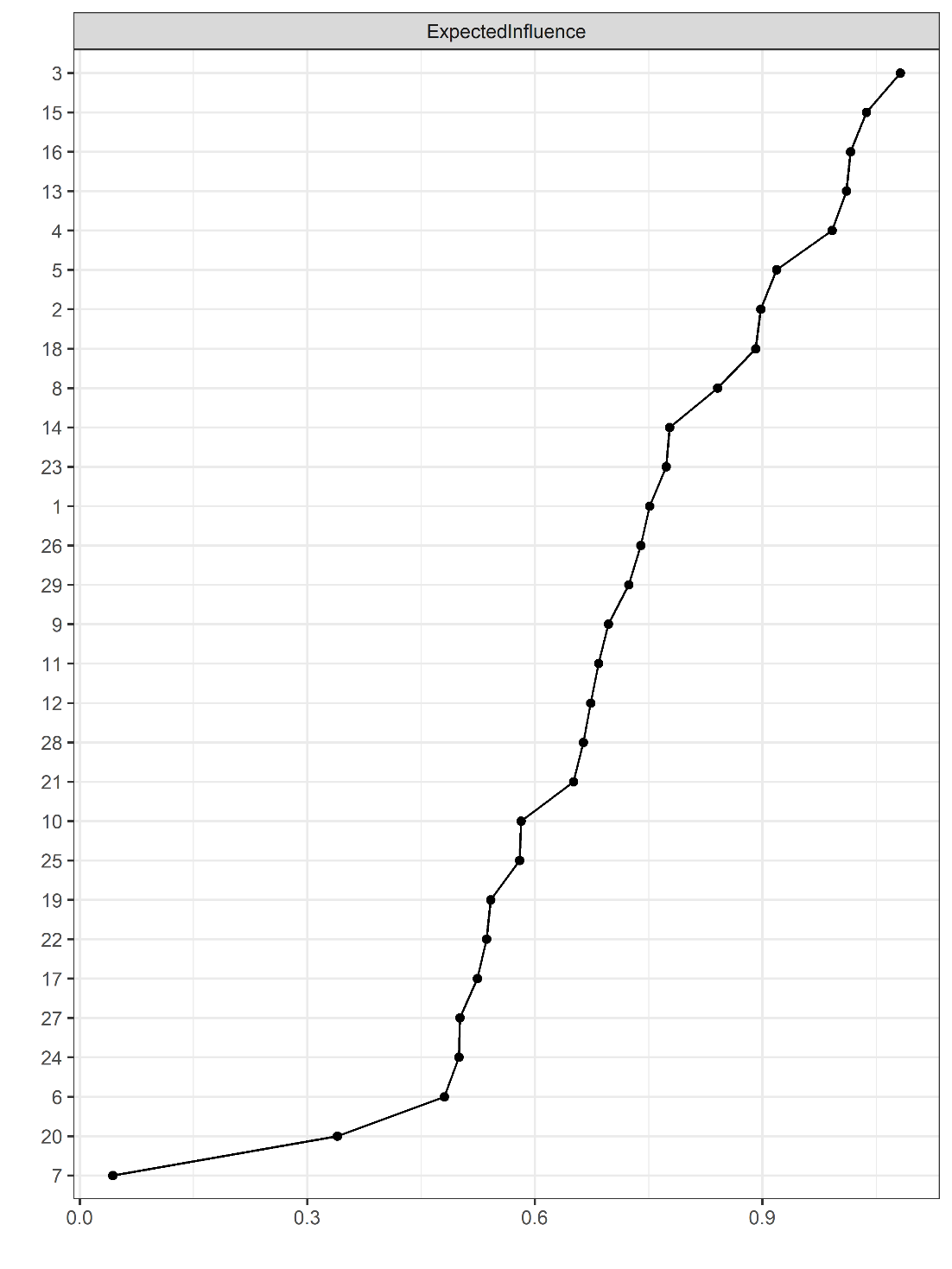
*

**Figure S2** *Bridge Centrality measure for the contemporaneous network.*


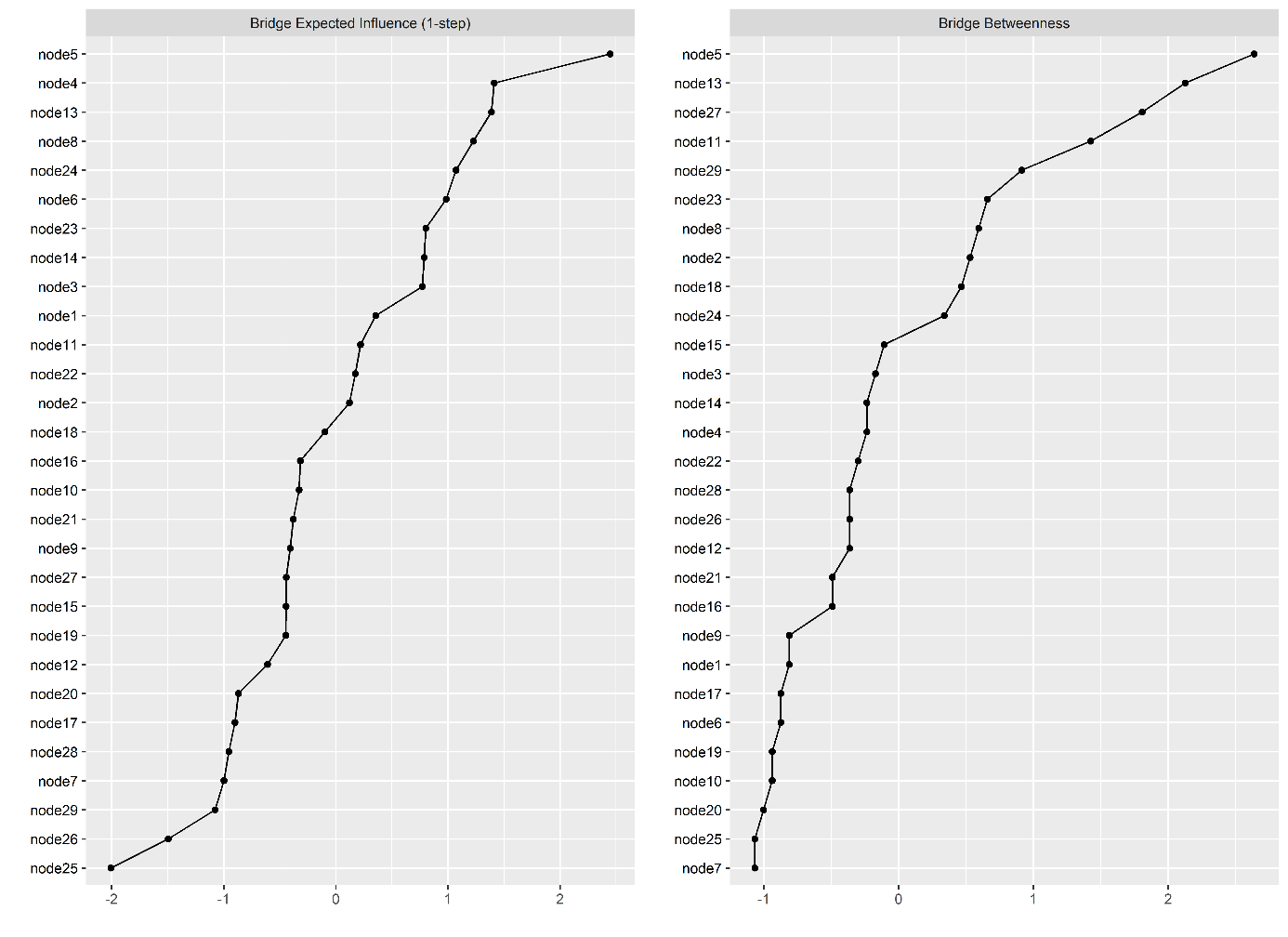


**Appendix S4: Temporal Network Analysis**

**Table S6** *In-strength and out-strength values for each node in the temporal network. In-strength reflects how strongly a node is predicted by other nodes, while out-strength indicates how strongly a node predicts other nodes over time. The difference (out-strength minus in-strength) highlights the relative dominance of a node in the network. Values close to zero indicate balanced in- and out-strength, suggesting the node is equally influenced by and influencing other symptoms. Negative values indicate higher out-strength, meaning the node exerts more influence on other nodes than it receives. Positive values indicate higher in-strength, meaning the node is more influenced by other nodes than it influences others.*

| Node | Variable | In-strength | Out-strength | Difference |
| --- | --- | --- | --- | --- |
| 1 | GAD 1 | 0.41 | 0.50 | -0.09 |
| 2 | GAD 2 | 0.44 | 1.35 | -0.90 |
| 3 | GAD 3 | 0.37 | 0.14 | 0.23 |
| 4 | GAD 4 | 0.37 | 0.21 | 0.16 |
| 5 | GAD 6 | 0.28 | 0.16 | 0.12 |
| 6 | SDQ 5 | 0.30 | 0.22 | 0.08 |
| 7 | SDQ 7 | 0.04 | 0.06 | -0.02 |
| 8 | PHQ 1 | 0.32 | 0.09 | 0.23 |
| 9 | PHQ 2 | 0.13 | 0.10 | 0.03 |
| 10 | PHQ 3 | 0.25 | 0.10 | 0.15 |
| 11 | PHQ 4 | 0.37 | 0.15 | 0.22 |
| 12 | PHQ 5 | 0.32 | 0.05 | 0.27 |
| 13 | PHQ 6 | 0.32 | 0.16 | 0.16 |
| 14 | PHQ 7 | 0.31 | 0.42 | -0.11 |
| 15 | CHEQ 4 | 0.58 | 1.09 | -0.50 |
| 16 | CHEQ 5 | 0.59 | 1.04 | -0.45 |
| 17 | CHEQ 7 | 0.44 | 0.33 | 0.10 |
| 18 | CHEQ 8 | 0.50 | 0.48 | 0.02 |
| 19 | SOCS 1 | 0.34 | 0.21 | 0.14 |
| 20 | SOCS 2 | 0.17 | 0.03 | 0.14 |
| 21 | SOCS 3 | 0.22 | 0.46 | -0.23 |
| 22 | SOCS 4 | 0.21 | 0.02 | 0.19 |
| 23 | SOCS 5 | 0.29 | 0.22 | 0.07 |
| 24 | CAPE 2 | 0.26 | 0.33 | -0.06 |
| 25 | ASRM 1 | 0.35 | 0.17 | 0.18 |
| 26 | ASRM 2 | 0.31 | 0.27 | 0.04 |
| 27 | ASRM 3 | 0.09 | 0.22 | -0.13 |
| 28 | ASRM 4 | 0.15 | 0.36 | -0.21 |
| 29 | ASRM 5 | 0.32 | 0.14 | 0.17 |

**Table S7** *Exact in-strength values for each node in the temporal network. Edges close to zero (<0.005) are not displayed for better clarity.*

| **PredictedNode** | **1** | **2** | **3** | **4** | **5** | **6** | **7** | **8** | **9** | **10** | **11** | **12** | **13** | **14** | **15** | **16** |
| --- | --- | --- | --- | --- | --- | --- | --- | --- | --- | --- | --- | --- | --- | --- | --- | --- |
| **1** | 0.11 | 0.18 |  |  | -0.01 |  |  |  | 0.02 |  |  |  |  |  |  | 0.07 |
| **2** | 0.07 | 0.23 | 0.12 |  |  |  |  |  |  |  |  |  |  |  |  |  |
| **3** | 0.00 | 0.27 |  | 0.06 |  |  |  |  |  |  |  |  |  |  |  | 0.05 |
| **4** | 0.10 | 0.15 |  |  |  | 0.04 |  |  |  |  |  |  |  |  |  |  |
| **5** |  | 0.11 |  |  | 0.09 |  |  |  |  |  |  |  |  |  |  |  |
| **6** |  |  | 0.01 | 0.05 |  | 0.11 |  |  |  |  | 0.06 |  |  | 0.06 |  |  |
| **7** |  |  |  |  |  |  | 0.06 |  |  |  |  |  | -0.05 | 0.05 |  |  |
| **8** |  | 0.16 |  |  |  |  |  | 0.11 |  |  |  |  |  |  |  |  |
| **9** |  |  |  |  |  |  |  |  |  |  |  |  | 0.08 |  |  |  |
| **10** |  | 0.06 |  |  |  |  |  |  |  | 0.10 |  |  |  | 0.05 |  | 0.04 |
| **11** |  |  |  |  |  | 0.06 |  |  |  |  | 0.07 |  |  |  |  | 0.12 |
| **12** |  |  | 0.02 |  | 0.06 |  |  |  | 0.01 |  |  | 0.08 | 0.07 |  |  |  |
| **13** | 0.03 | 0.12 |  |  | -0.03 |  |  |  |  |  |  |  | 0.07 |  | 0.13 |  |
| **14** |  |  |  | 0.07 |  |  |  |  |  |  |  |  |  | 0.11 | 0.05 |  |
| **15** |  |  |  | 0.02 |  |  |  |  |  |  |  |  |  |  | 0.30 | 0.18 |
| **16** |  |  |  | 0.02 |  |  |  |  |  |  |  |  |  | 0.02 | 0.28 | 0.17 |
| **17** |  |  |  |  |  |  |  |  |  |  |  |  |  |  | 0.13 | 0.15 |
| **18** |  | 0.06 | -0.02 |  |  |  |  |  |  |  |  |  |  |  | 0.16 | 0.16 |
| **19** | 0.10 |  |  |  | 0.05 |  |  | -0.01 | 0.06 |  |  |  |  |  |  |  |
| **20** |  |  |  |  |  |  |  |  |  |  |  |  | -0.05 |  |  | 0.07 |
| **21** | 0.03 |  |  |  |  |  |  |  |  |  |  |  |  |  |  | 0.04 |
| **22** |  |  |  |  |  |  |  |  |  |  |  |  |  | 0.07 |  |  |
| **23** | 0.06 | 0.05 |  |  |  |  |  |  | 0.03 |  |  |  |  |  |  |  |
| **24** |  |  |  |  |  |  |  |  |  |  |  |  |  |  | 0.08 |  |
| **25** |  |  | 0.01 |  |  |  |  |  |  |  |  |  |  |  |  |  |
| **26** |  | -0.03 |  |  |  |  |  |  |  |  |  |  | 0.05 |  | -0.04 |  |
| **27** |  |  |  |  |  |  |  |  | -0.02 |  |  | -0.04 |  | 0.07 |  |  |
| **28** |  |  |  |  |  |  |  |  |  |  |  |  |  |  |  |  |
| **29** |  |  |  |  |  |  |  |  |  |  | 0.02 |  |  |  |  |  |

| **PredictedNode** | **17** | **18** | **19** | **20** | **21** | **22** | **23** | **24** | **25** | **26** | **27** | **28** | **29** |
| --- | --- | --- | --- | --- | --- | --- | --- | --- | --- | --- | --- | --- | --- |
| **1** |  |  |  |  | 0.03 |  |  |  |  |  |  | 0.02 |  |
| **2** |  |  |  |  | 0.04 | -0.01 |  |  |  |  |  |  |  |
| **3** |  |  |  |  |  |  |  |  |  |  |  |  |  |
| **4** |  |  |  |  |  |  |  |  |  |  | 0.05 | 0.02 |  |
| **5** |  | 0.09 |  |  |  |  |  |  |  |  |  |  |  |
| **6** |  |  |  |  |  |  |  |  |  |  |  |  |  |
| **7** |  |  |  |  |  |  |  |  |  |  |  |  | -0.02 |
| **8** |  | 0.07 |  |  |  |  |  |  |  |  |  |  | -0.02 |
| **9** |  | 0.07 |  |  |  | -0.01 |  |  |  |  |  |  |  |
| **10** |  |  |  |  |  |  |  |  |  |  |  |  |  |
| **11** |  | 0.08 |  |  |  |  |  | 0.05 |  |  |  |  |  |
| **12** |  | 0.08 |  |  |  |  |  |  |  |  |  |  |  |
| **13** |  |  |  |  |  |  |  |  |  |  |  |  |  |
| **14** |  |  |  |  | 0.04 |  | 0.04 |  |  |  |  |  |  |
| **15** | 0.09 |  |  |  |  |  |  |  |  |  |  |  |  |
| **16** | 0.10 |  |  |  | 0.02 | -0.03 |  |  |  |  |  |  |  |
| **17** | 0.08 |  |  |  | 0.03 |  | 0.04 |  |  |  |  |  |  |
| **18** |  | 0.09 | 0.03 |  | 0.02 | -0.02 |  | 0.03 |  |  |  |  |  |
| **19** |  |  | 0.10 |  | 0.06 |  |  |  |  |  |  |  |  |
| **20** |  |  |  | 0.13 | 0.04 |  |  |  |  | -0.02 |  |  |  |
| **21** |  |  | 0.07 |  | 0.12 |  |  |  |  |  |  |  | -0.03 |
| **22** |  |  | 0.05 |  |  | 0.09 |  |  |  |  |  |  |  |
| **23** | 0.06 |  |  |  | 0.04 |  | 0.08 |  |  |  | -0.04 |  |  |
| **24** |  |  |  |  |  |  |  | 0.18 |  |  |  |  |  |
| **25** |  |  |  |  |  |  |  |  | 0.17 |  | 0.06 |  | 0.11 |
| **26** |  |  |  | -0.03 |  |  |  |  |  | 0.19 |  | 0.07 | 0.10 |
| **27** |  |  |  | -0.07 |  |  |  |  |  |  | 0.14 |  |  |
| **28** |  |  |  |  |  |  |  |  |  |  |  | 0.15 |  |
| **29** |  |  | -0.03 |  |  |  | 0.06 | 0.06 |  | 0.10 |  | 0.10 |  |

**Table S8** *Exact out-strength values for each node in the temporal network. Edges close to zero (<0.005) are not displayed for better clarity.*

| **Predictor Node** | **1** | **2** | **3** | **4** | **5** | **6** | **7** | **8** | **9** | **10** | **11** | **12** | **13** | **14** | **15** | **16** |
| --- | --- | --- | --- | --- | --- | --- | --- | --- | --- | --- | --- | --- | --- | --- | --- | --- |
| **1** | 0.11 | 0.07 |  | 0.10 |  |  |  |  |  |  |  |  | 0.03 |  |  |  |
| **2** | 0.18 | 0.23 | 0.27 | 0.15 | 0.11 |  |  | 0.16 |  | 0.06 |  |  | 0.12 |  |  |  |
| **3** |  | 0.12 |  |  |  | 0.01 |  |  |  |  |  | 0.02 |  |  |  |  |
| **4** |  |  | 0.06 |  |  | 0.05 |  |  |  |  |  |  |  | 0.07 | 0.02 | 0.02 |
| **5** | -0.01 |  |  |  | 0.09 |  |  |  |  |  |  | 0.06 | -0.03 |  |  |  |
| **6** |  |  |  | 0.04 |  | 0.11 |  |  |  |  | 0.06 |  |  |  |  |  |
| **7** |  |  |  |  |  |  | 0.06 |  |  |  |  |  |  |  |  |  |
| **8** |  |  |  |  |  |  |  | 0.11 |  |  |  |  |  |  |  |  |
| **9** | 0.02 |  |  |  |  |  |  |  |  |  |  | 0.01 |  |  |  |  |
| **10** |  |  |  |  |  |  |  |  |  | 0.10 |  |  |  |  |  |  |
| **11** |  |  |  |  |  | 0.06 |  |  |  |  | 0.07 |  |  |  |  |  |
| **12** |  |  |  |  |  |  |  |  |  |  |  | 0.08 |  |  |  |  |
| **13** |  |  |  |  |  |  | -0.05 |  | 0.08 |  |  | 0.07 | 0.07 |  |  |  |
| **14** |  |  |  |  |  | 0.06 | 0.05 |  |  | 0.05 |  |  |  | 0.11 |  | 0.02 |
| **15** |  |  |  |  |  |  |  |  |  |  |  |  | 0.13 | 0.05 | 0.30 | 0.28 |
| **16** | 0.07 |  | 0.05 | 0.00 |  |  |  |  |  | 0.04 | 0.12 |  |  |  | 0.18 | 0.17 |
| **17** |  |  |  |  |  |  |  |  |  |  |  |  |  |  | 0.09 | 0.10 |
| **18** |  |  |  |  | 0.09 |  |  | 0.07 | 0.07 |  | 0.08 | 0.08 |  |  |  |  |
| **19** |  |  |  |  |  |  |  |  |  |  |  |  |  |  |  |  |
| **20** |  |  |  |  |  |  |  |  |  |  |  |  |  |  |  |  |
| **21** | 0.03 | 0.04 |  |  |  |  |  |  |  |  |  |  |  | 0.04 |  | 0.02 |
| **22** |  | -0.01 |  |  |  |  |  |  | -0.01 |  |  |  |  |  |  | -0.03 |
| **23** |  |  |  |  |  |  |  |  |  |  |  |  |  | 0.04 |  |  |
| **24** |  |  |  |  |  |  |  |  |  |  | 0.05 |  |  |  |  |  |
| **25** |  |  |  |  |  |  |  |  |  |  |  |  |  |  |  |  |
| **26** |  |  |  |  |  |  |  |  |  |  |  |  |  |  |  |  |
| **27** |  |  |  | 0.05 |  |  |  |  |  |  |  |  |  |  |  |  |
| **28** | 0.02 |  |  | 0.02 |  |  |  |  |  |  |  |  |  |  |  |  |
| **29** |  |  |  |  |  |  | -0.02 | -0.02 |  |  |  |  |  |  |  |  |

| **Predictor Node** | **17** | **18** | **19** | **20** | **21** | **22** | **23** | **24** | **25** | **26** | **27** | **28** | **29** |
| --- | --- | --- | --- | --- | --- | --- | --- | --- | --- | --- | --- | --- | --- |
| **1** |  |  | 0.10 |  | 0.03 |  | 0.06 |  |  |  |  |  |  |
| **2** |  | 0.06 |  |  |  |  | 0.05 |  |  | -0.03 |  |  |  |
| **3** |  | -0.02 |  |  |  |  |  |  | 0.01 |  |  |  |  |
| **4** |  |  |  |  |  |  |  |  |  |  |  |  |  |
| **5** |  |  | 0.05 |  |  |  |  |  |  |  |  |  |  |
| **6** |  |  |  |  |  |  |  |  |  |  |  |  |  |
| **7** |  |  |  |  |  |  |  |  |  |  |  |  |  |
| **8** |  |  | -0.01 |  |  |  |  |  |  |  |  |  |  |
| **9** |  |  | 0.06 |  |  |  | 0.03 |  |  |  | -0.02 |  |  |
| **10** |  |  |  |  |  |  |  |  |  |  |  |  |  |
| **11** |  |  |  |  |  |  |  |  |  |  |  |  | 0.02 |
| **12** |  |  |  |  |  |  |  |  |  |  | -0.04 |  |  |
| **13** |  |  |  | -0.05 |  |  |  |  |  | 0.05 |  |  |  |
| **14** |  |  |  |  |  | 0.07 |  |  |  |  | 0.07 |  |  |
| **15** | 0.13 | 0.16 |  |  |  |  |  | 0.08 |  | -0.04 |  |  |  |
| **16** | 0.15 | 0.16 |  | 0.07 | 0.04 |  |  |  |  |  |  |  |  |
| **17** | 0.08 |  |  |  |  |  | 0.06 |  |  |  |  |  |  |
| **18** |  | 0.09 |  |  |  |  |  |  |  |  |  |  |  |
| **19** |  | 0.03 | 0.10 |  | 0.07 | 0.05 |  |  |  |  |  |  | -0.03 |
| **20** |  |  |  | 0.13 |  |  |  |  |  | -0.03 | -0.07 |  |  |
| **21** | 0.03 | 0.02 | 0.06 | 0.04 | 0.12 |  | 0.04 |  |  |  |  |  |  |
| **22** |  | -0.02 |  |  |  | 0.09 |  |  |  |  |  |  |  |
| **23** | 0.04 |  |  |  |  |  | 0.08 |  |  |  |  |  | 0.06 |
| **24** |  | 0.03 |  |  |  |  |  | 0.18 |  |  |  |  | 0.06 |
| **25** |  |  |  |  |  |  |  |  | 0.17 |  |  |  |  |
| **26** |  |  |  | -0.02 |  |  |  |  |  | 0.19 |  |  | 0.10 |
| **27** |  |  |  |  |  |  | -0.04 |  | 0.06 |  | 0.14 |  |  |
| **28** |  |  |  |  |  |  |  |  |  | 0.07 |  | 0.15 | 0.10 |
| **29** |  |  |  |  | -0.03 |  |  |  | 0.11 | 0.10 |  |  |  |
